# Atmospheric pressure chemical vapor deposition growth of vertically aligned SnS_2_ and SnSe_2_ nanosheets†

**DOI:** 10.1039/d1ra05672g

**Published:** 2021-11-11

**Authors:** Ayrton Sierra-Castillo, Emile Haye, Selene Acosta, Raul Arenal, Carla Bittencourt, Jean-François Colomer

**Affiliations:** Research Group on Carbon Nanostructures (CARBONNAGe), University of Namur 5000 Namur Belgium ayrton.sierracastillo@gmail.com; Laboratoire d'Analyse par Réactions Nucléaires (LARN), Namur Institute of Structured Matter (NISM), University of Namur 5000 Namur Belgium; Chimie des Interactions Plasma–Surface (ChIPS), Research Institute for Materials Science and Engineering, Université de Mons 7000 Mons Belgium; Instituto de Nanociencia y Materiales de Aragon (INMA), CSIC-Universidad de Zaragoza 50009 Zaragoza Spain; Laboratorio de Microscopias Avanzadas (LMA), Universidad de Zaragoza 50018 Zaragoza Spain; ARAID Foundation 50018 Zaragoza Spain

## Abstract

Laminated metal dichalcogenides are candidates for different potential applications ranging from catalysis to nanoelectronics. However, efforts are still needed to optimize synthesis methods aiming to control the number of layers, morphology, and crystallinity, parameters that govern the properties of the synthesized materials. Another important parameter is the thickness and the length of the samples with the possibility of large-scale growth of target homogeneous materials. Here, we report a chemical vapor deposition method at atmospheric pressure to produce vertically aligned tin dichalcogenide based-materials. Tin disulfide (SnS_2_) and tin diselenide (SnSe_2_) vertically aligned nanosheets have been synthesized and characterized by different methods showing their crystallinity and purity. Homogenous crystalline 2H-phase SnS_2_ nanosheets with high purity were synthesized with vertical orientation on substrates; sulfur vacancies were observed at the edges of the sheets. Similarly, in the crystalline 2H phase SnSe_2_ nanosheets selenium vacancies were observed at the edges. Moreover, these nanosheets are larger than the SnS_2_ nanosheets, show lower nanosheet homogeneity on substrates and contamination with selenium atoms from the synthesis was observed. The synthesized nanomaterials are interesting in various applications where the edge accessibility and/or directionality of the nanosheets play a major role as for example in gas sensing or field emission.

## Abstract 2

SnS_2_ and SnSe_2_ nanosheets were synthesized vertically aligned in different substrates by an AP-CVD method.

## Graphical abstract

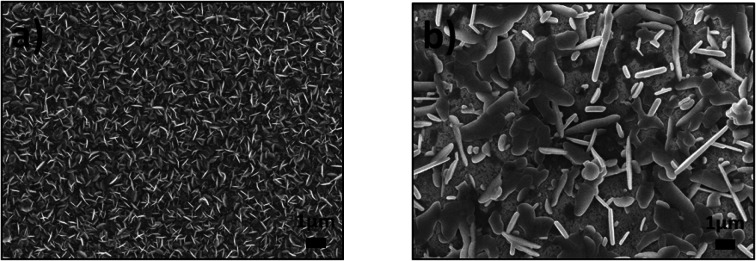

## Introduction

Two-dimensional (2D) layered nanostructures have received increasing interest due to their unique nanoscale phenomena and their potential applications ranging from electronics and energy to catalysis. In the last decade, different 2D layered dichalcogenides, such as molybdenum disulfide (MoS_2_),^1^ tungsten disulfide (WS_2_),^2^ molybdenum diselenide (MoSe_2_),^3^ tungsten diselenide (WSe_2_),^4^ tin disulfide (SnS_2_)^5^ and tin diselenide (SnSe_2_)^6^ have been widely studied. The interest in these nanomaterials was trigged by their potential applications in field-effect transistors (FETs),^7^ photodetectors,^8^ solar cells,^9,10^ and gas sensors.^11,12^ SnS_2_ and SnSe_2_ have a similar configuration as MoS_2_, in which a Sn layer is sandwiched between two S or Se layers to form a “three-layer” structure (one metal atom between two chalcogenide atoms, covalently bonded, and the layers are linked between them by van der Waals forces).

Tin disulfide (SnS_2_) and tin diselenide (SnSe_2_) belong to the family IVA–VIA group, which are earth-abundant and environment-friendly materials, and due to their low cost, they present a considerable advantage for nanoelectronics and optoelectronics. There are several recently reports on the optical, electrical, and photoelectric properties of SnS_2_ nanomaterials.^13,14^ SnS_2_ is a broadband gap semiconductor with band energy gap of 2.2 eV.^14^ In addition, SnS_2_ has a higher theoretical capacity, compared to other 2D materials, such as MoS_2_ and WS_2_.^15^ All these properties make SnS_2_ a promising candidate for FETs, integrated logic circuits, photodetectors; energy storage materials and solar cells. With a direct band gap of 1.4 eV,^15^ SnSe_2_ is attractive in the fields of nanoelectronics and optoelectronics, but also due to its structure and low cost, it is a strong candidate for sensing applications.^16^

Therefore, the controllable growth of these materials is the first and critical step for their device applications, to have a reproductible control of the structure, including the number of layers, the crystallographic phase composition, or/and film morphology. Previous studies have already demonstrated that these 2D materials can be prepared using chemical^17^ or mechanical^18^ exfoliation, spray pyrolysis,^19^ thermal evaporation,^20^ hydrothermal method^12,21,22^ and chemical vapor deposition (CVD).^23^ In general, the research in this area focus on the synthesis of monolayer domains of controlled size, whose properties are interesting for applications in electronics.^24^ CVD has proven to be an effective method for synthesizing this type of material where control of the number of layers is important, however CVD also allows the control of the material morphology opening up new applications. Previously with CVD, high-quality 2D layered nanostructures, such as MoS_2_ (ref. 25) and WSe_2_ (ref. 26) were synthesized vertically aligned to the substrate, with possible applications as gas sensors^27^ or field emission sources.^28^

In the CVD method, different experimental geometries and strategies can be used leading to nanomaterials with different morphology, crystallinity (phase), crystal size, *etc.*^29^ This is also true for the CVD growth of metal dichalcogenides, here taking advantage of this unique characteristic of the CVD growth, the focus is done on tin dichalcogenide materials. The CVD strategy used is based on the co-evaporation of two precursors one for the metal, the other for the chalcogenide. Therefore, using sulfur and tin powers for the synthesis of SnS_2_ hexagonal nanosheets are obtained.^30^ While, when sulfur and SnS_2_ powder, hexagonal and truncated triangular nanosheets are observed.^31^ These latter examples concern the in-plane growth of the 2D materials, however other morphologies have been reported also based on CVD with co-evaporation. As well, vertically aligned SnS_2_ nanosheets with the hexagonal 2H phase, have been synthesized using S and SnI_2_ powder^5^ or SnCl_4_·5H_2_O (ref. 32) as tin source. These same strategies were used to produce Se-based nanomaterials, for in-plane growth, using Se with SnI_2_, few-layers SnSe_2_ were formed on mica substrate with triangular^33^ or hexagonal^34^ crystal shapes. Using different sources, *i.e.* Se and SnO_2_ powder hexagonal crystals were obtained.^35^ Within this perspective, using SnSe as tin precursor, the crystallographic phase was fine controlled by tuning the control by synthesis temperature.^36^ Furthermore, using diethethyl selenide with SnCl_4_ as precursor for the synthesis, a vertical preferential growth of SnSe_2_ was observed.^37^

A different CVD strategy for the synthesis of metal dichalcogenides is direct sulfurization^27^ or selenization^38^ of metal films. The deposition of a metallic film of controlled thickness allows to know precisely where the nanocrystals will be synthesized, avoiding non-uniform dispersion and distribution of the nanosheets in the case of evaporation of metal precursors with chalcogenide as explained previously.^39^ Another advantage of this strategy is the possibility to control the nanosheets height – when they are vertically aligned – with the well-defined film thickness.^40^ However, it is difficult, if not impossible, to synthesize monolayer crystal domains using this strategy because of the adapted thickness of the film and the high temperature used causing the film dewetting. Finally, for the synthesis of vertically aligned nanosheets, this strategy seems to be a reproducible and can be used in the synthesis of many metal dichalcogenides-based materials.^27,38,40^ The metallic films with controlled thickness can be deposited using different physical methods such as e-beam evaporation,^27^ magnetron sputtering,^41^*etc.* In this work, we propose to apply this strategy for the first time to the best of our knowledge for the synthesis of vertically-aligned SnS_2_ and SnSe_2_ by rapid sulfurization and selenization, respectively, using the atmospheric pressure CVD (APCVD) technique. The structural, morphological and chemical characterization of the nanostructures, performed by X-ray diffraction, electron microscopies, Raman and X-ray photoelectron spectroscopies confirm the crystallinity and purity of the materials.

## Materials and methods

### Materials

The commercial products, namely the Sn target (purity 99.95%) was purchased from Micro to Nano, S (purity 99%) and Se powder (purity 99%) were purchased from Alfa Aesar, all the products were used as received.

### Synthesis of SnS_2_ and SnSe_2_ nanosheets

The SnS_2_ and SnSe_2_ nanosheets were synthesized *via* atmospheric pressure CVD. A 50 nm thick Sn film was deposited on a sapphire substrate by direct current magnetron sputtering (sputter current 100 mA), using a commercial sputter deposition system (Quorum Q15T/ES) in an Argon (99.9995%) atmosphere. A pure Sn target with a 57 mm diameter was used, and the substrates were placed on a rotating holder with a 90 mm target. The pressure of the argon in the deposition chamber was 1 × 10^−3^ mbar. The Sn film thickness was monitored using a quartz microbalance mounted in the deposition chamber system.

Before the synthesis, the Sn film was oxidized at 300 °C with air flow using atmospheric pressure. The influence of the Sn oxidation is presented in the Fig. S1.† The sulfurization was performed on a quartz tube (reactor). Firstly, the Sn film on the sapphire substrate was introduced into the reactor with the S powder, and this was placed in two predefined zones of the tube (0.210 g was used in each zone) in order to be in the correct temperature zones when the reactor was inserted into the furnace. The S powder was used without any further purification. The reactor was flushed for one hour to remove (reduce the amount of) oxygen, using a 0.745 L min^−1^ argon flow outside of the furnace, prior to the sulfurization process. After that, the reactor was put inside of the furnace and the argon flow reduced to 0.070 L min^−1^. As in a typical sulfurization process, the reactor is moved in order to place the S powder at 550 °C into the oven, another S powder is placed close to the Sn film sample at the same right temperature zone, with the argon flow for 30 minutes (Fig. S2†). After the reaction, the quartz tube was removed from the reactor and was cooled with the argon flow for 1 h. In Fig. S3 and S4† different synthesis temperatures and gas flows are presented to show the effect of changing conditions for the synthesis of our material, respectively.

For the second material, a selenization was performed following the previous details described for sulfurization. However, instead of S, we used Se powder (0.350 g was used in each zone). The reactor was flushed for one hour to remove (reduce) oxygen, using a 0.745 L min^−1^ argon flow outside of the furnace, prior to the sulfurization process. After that, the reactor was put inside of the furnace and the argon flow changed to 0.010 L min^−1^. A 0.010 L min^−1^ H_2_ flow was inserted during this synthesis to help with the reaction. The presence of a strong reducer, such as the H_2_, is mandatory for the selenization reaction, in contrast with the sulfurization reaction.^26^ In a typical selenization process and as in the sulfurization reaction, the reactor is moved in order to place the Se powder at 450 °C into the oven, another Se powder is placed close to the Sn film sample at the same right temperature zone, with the argon and hydrogen flow for 30 minutes (Fig. S5†). After the reaction, the H_2_ flow was stopped, and the quartz tube was removed from the reactor and was cooled with the argon flow for 1 h. Similar as in the case of the first material, Fig. S6 and S7† show the influence of the synthesis temperature and gas flow during the synthesis process.

### Characterization techniques

Scanning electron microscopy (SEM) analyses were conducted on a JEOL 7500F microscope operating at 15 kV. Transmission electron microscopy studies were carried out on a TECNAI T20 microscope working under 200 kV for bright-field images and electron diffraction and on an aberration-corrected FEI Titan Low-base microscope for scanning TEM. This STEM instrument was operated at 80 kV and is equipped with an Oxford silicon-drift-detector (SDD) for X-ray spectroscopic (EDS) analyses. The SnS_2_ and SnSe_2_ nanosheets were examined using a micro-Raman system (Senterra Bruker Optik GmbH) with a 3 cm^−1^ resolution, using a laser excitation laser source (532 nm wavelength), and a laser power of 2 and 5 mW, respectively. The structure of the sample has been investigated by X-ray diffraction (XRD) using a Panalytical X'Pert PRO diffractometer (Cu Kα radiation, Bragg–Brentano geometry). The chemistry of the SnS_2_ and SnSe_2_ nanosheets was studied with X-ray photoelectron spectroscopy (XPS) using an Escalab 250i Thermo Fisher Scientific™ instrument (consisting of a monochromatic Al Kα X-ray source at 1486.68 eV and working at 13.9 keV and 5.2 mA). The base pressure during spectra acquisition was better than 10^10^ mbar. The O 1s, Sn 3d, S 2p and Se 3d core level spectra are recorded with a pass energy of 20 eV, with 20 scans, in sequential mode, with a spot size of 250 × 250 μm^2^. The calibration and linearity of the binding energy scale is regularly determined by Cu 2p_3/2_, Ag 3d_5/2_ and Au 4f_7/2_ energies. With the selected scan parameters and a pass energy of 25 eV, the energy resolution was ≈0.7 eV measured on the FWHM of the Ag 3d_5/2_. A flood gun is used during the analysis to limit the charge shifting, which has been further checked by the position of the valence band minimum. No charge shift is applied to the spectra. The authors are aware of the recent warnings about XPS analysis on insulating samples.^42–44^

## Results

**Fig. 1:**
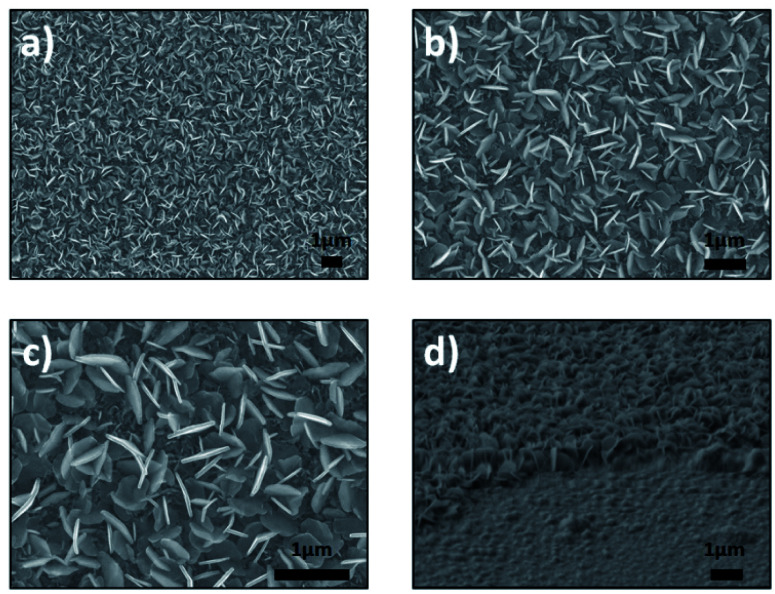
SEM images of synthesized products for vertically aligned SnS_2_ nanosheets, (a–c) top view and (d) cross section (62°).

**Fig. 2:**
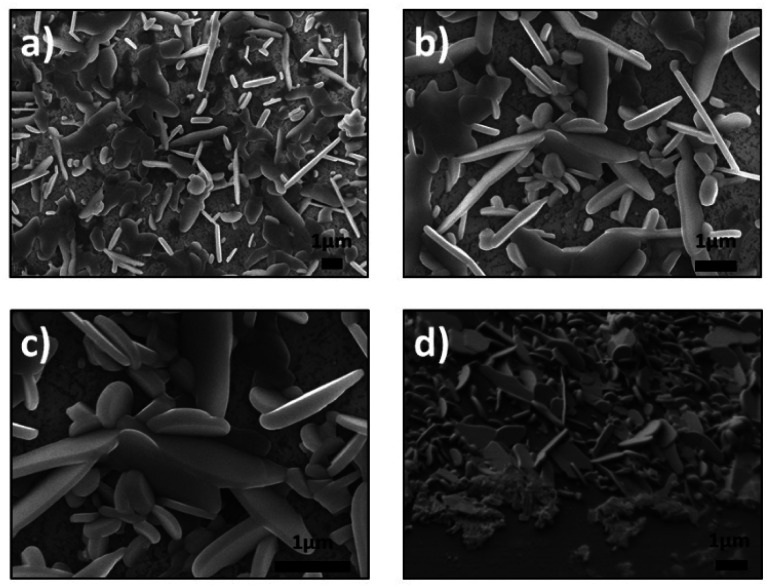
SEM images of synthesized products for vertically aligned SnSe_2_ nanosheets, (a–c) top view and (d) cross section (45°).

Fig. 1 and 2 show the SEM images recorded after the sulfurization and selenization of 50 nm of Sn deposited on sapphire substrate. For the two different materials, SnS_2_ and SnSe_2_ nanosheets can be observed. The low-magnification images in Fig. 1a and b reveal a large amount of SnS_2_ nanoplatelets that are uniformly distributed on the sapphire substrate. The high magnification image (Fig. 1c) shows SnS_2_ nanoplatelets, with an average length of 500–600 nm and a thickness of 30–35 nm, have a well-defined shape with sharp edges. The difference between these three images is the magnification. The homogeneity of the sample (Fig. 1a) can be observed (Fig. 1b and c). Fig. 1d shows the cross section of the sample. The image was taken at till angle of 62°, the presence of SnS_2_ nanosheets with vertical orientation can be observed.

Fig. 2a and b show the SnSe_2_ nanosheets with different orientations (vertical and horizontal). In addition, these nanosheets present a variety of sizes and shapes. This is not the case for the SnS_2_ samples, which are more homogeneous. The high magnification of Fig. 2c reveals the average length of the SnSe_2_ nanosheets (∼2.5 μm) as well as their thickness (ranging between 50 and 100 nm). The cross section of the sample at till angle of 45° confirms the different orientations of the SnSe_2_ nanosheets (Fig. 2d).

**Fig. 3:**
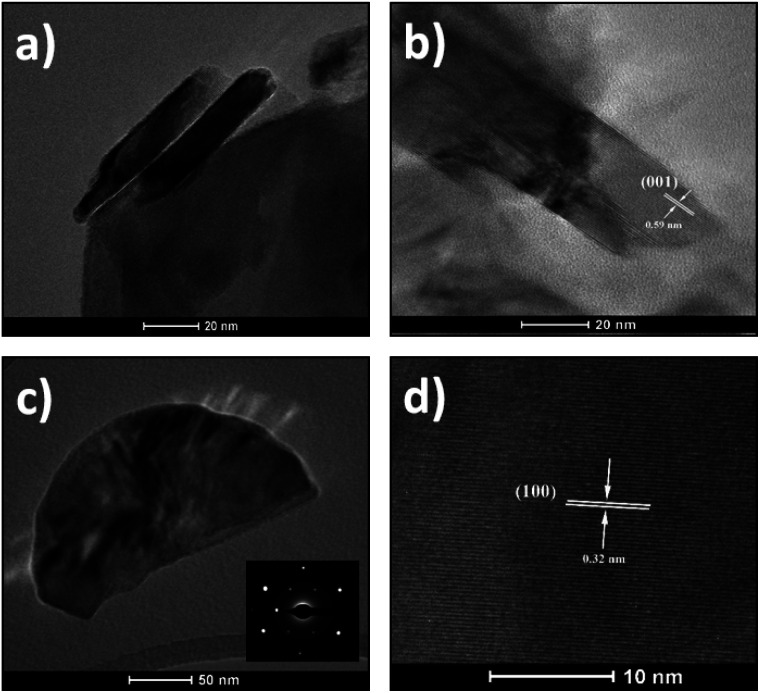
(a and b) TEM images of layered structures of SnS_2_ nanosheets. (c) Low magnification image of a single nanosheet with SAED pattern in inset and (d) high-resolution TEM image of the crystal showing the (001) crystal planes.

**Fig. 4:**
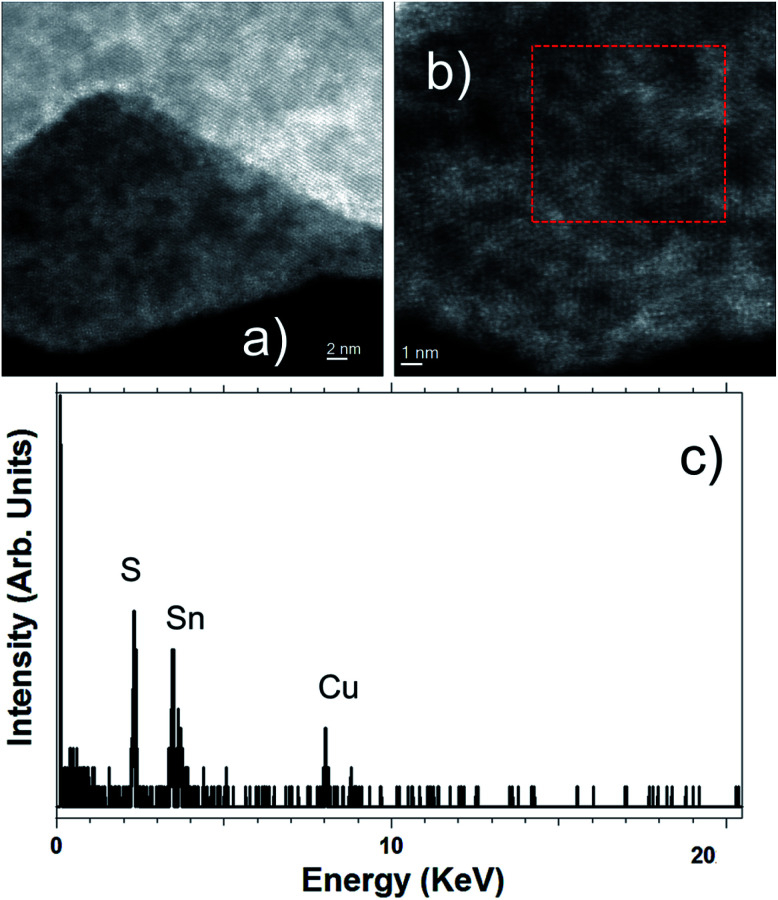
(a and b) HAADF-STEM images of SnS_2_ nanosheets. (c) EDS spectrum recorded in the red highlighted area of Fig. 3b.

For further information on morphology, the as-synthesized SnS_2_ nanosheets were characterized using TEM (Fig. 3 and 4). The Fig. 3a and b shows representative images of nanosheets exhibiting the layered structure with a typical interlayer distance of 0.59 nm corresponding to the (001) crystal planes. Fig. 3c shows a low magnification TEM image of a single nanosheet with the corresponding selected area diffraction (SAED) pattern in the inset. This SAED pattern exhibits well-defined and sharp spots in a hexagonal geometry, indicating a hexagonal crystal structure and the single-crystal nature of the observed nanosheet. A magnified region of this nanosheet is displayed in Fig. 3d. The lattice fringes with spacing to 0.32 nm are clearly observed and they correspond to the (100) crystal planes. This distance confirms also the hexagonal phase of the nanosheets.^45,46^

Fig. 4a and b show two high-angle annular dark-field (HAADF) STEM micrographs of two different SnS_2_ nanosheets, revealing the high crystallinity of these flakes. EDS analyses performed on these materials confirm the high purity of these flakes and the ratio of 2 for S/Sn (see Fig. 4c).

**Fig. 5:**
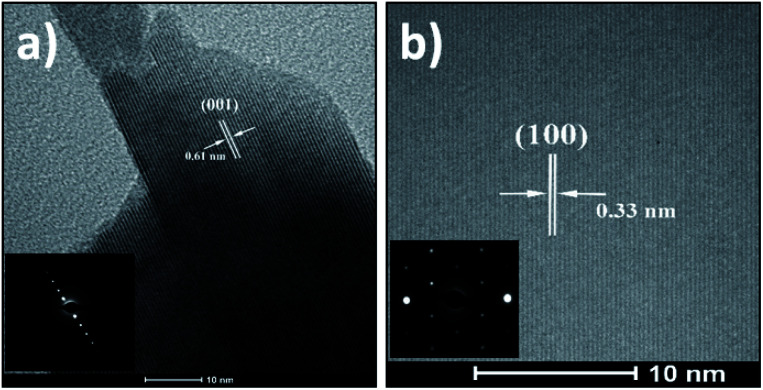
(a) TEM image of layered structures of nanosheet with the corresponding SAED pattern in inset and (b) high resolution image of the crystal showing the (001) crystal planes with in inset a SAED pattern of single nanosheet showing hexagonal spot symmetry.

**Fig. 6:**
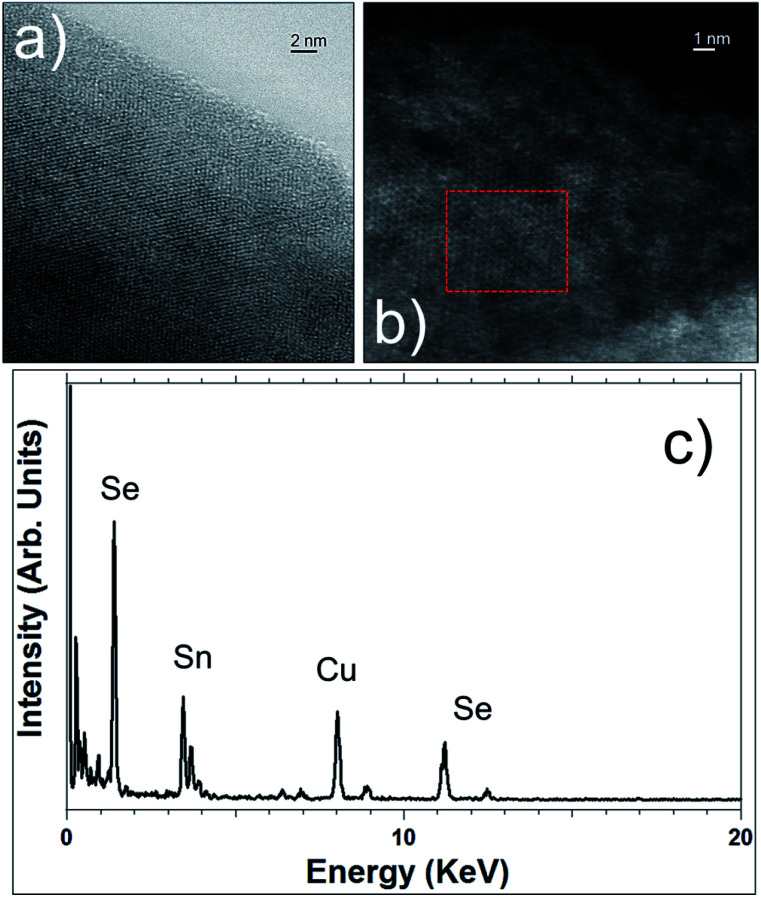
STEM images in bright-field mode (a) and in HAADF mode (b) of SnSe_2_ nanosheets. (c) EDS spectrum recorded in the red highlighted area of Fig. 6b.

Concerning the SnSe_2_ nanosheets, they have been characterized by TEM in the same way as the SnS_2_ nanosheets and the results are shown in Fig. 5 and 6. The layered structure of nanosheets can be observed in Fig. 5a with an interlayer spacing of 0.61 nm, which matches to the lattice spacing of (001) planes of hexagonal SnSe_2_ (ref. 47) (the corresponding SAED pattern in inset Fig. 5a is consistent with HRTEM image). Fig. 5b presents a high-resolution TEM image of nanosheet with well visible lattice fringes with a spacing of 0.33 nm that can be assigned to the (100) planes of the SnSe_2_. The SAED pattern is in inset of a single nanosheet in the (001) direction confirms the formation of the hexagonal phase of SnSe_2_ and the crystal structure.^48^

STEM imaging, bright-field and in HAADF modes (see Fig. 6), corroborates the high crystallinity of these SnSe_2_ nanosheets and their hexagonal structure. EDS (Fig. 6c) also confirms the SnSe_2_ chemical composition of those flakes and the ratio of 2 for Se/Sn.

**Fig. 7:**
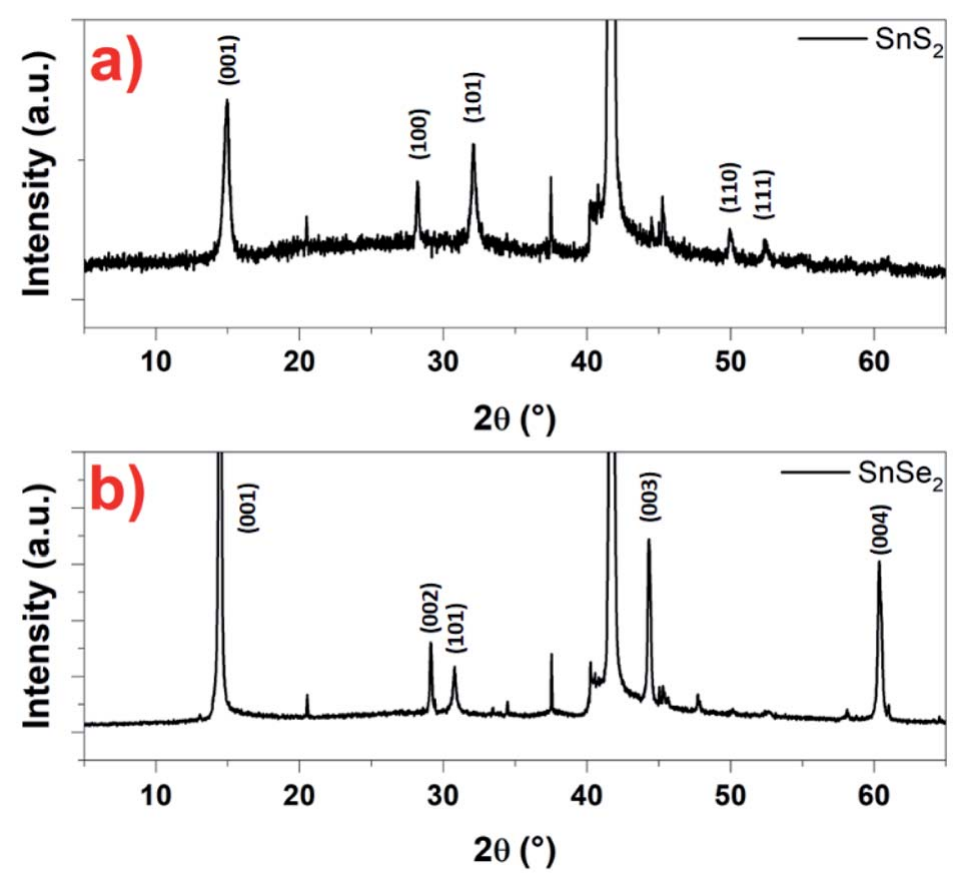
XRD pattern recorded on: (a) SnS_2_ and (b) SnSe_2_ nanosheets, respectively.

Based on the microscopy studies, the conclusions that can be drawn are that the nanosheets, whether SnS_2_ or SnSe_2_, are crystallized in a hexagonal structure. This has been checked using XRD. Indeed, in Fig. 7a, a typical XRD pattern of the as-prepared SnS_2_ nanosheets is shown. The diffraction peaks appearing at 14.5°, 28.6°, 32.3°, 50.2° and 52.1° are distinctly indexed to the (001), (100), (101), (110) and (111) planes of the hexagonal SnS_2_ phase, which are very close to the reported data (JCPDS card 23-0677) for the hexagonal 2H phase.^49^ In the same way, the typical XRD pattern of the so-prepared SnSe_2_ nanosheets in Fig. 7b show that are crystalline with a good match with the reported pattern for the hexagonal SnSe_2_ phase (JCPDS card 023-0602).^37^ Indeed, the diffraction peaks appearing at 14.5°, 29.05°, 30.7°, 44.3° and 60.3° are distinctly indexed to the (001), (002), (101), (003) and (004) planes. The peaks observed indicate the high purity and crystallinity of the products. For both samples, the intense diffraction peak around 42° corresponds to the (0001) plane of the substrate, in this case, the sapphire.

**Fig. 8:**
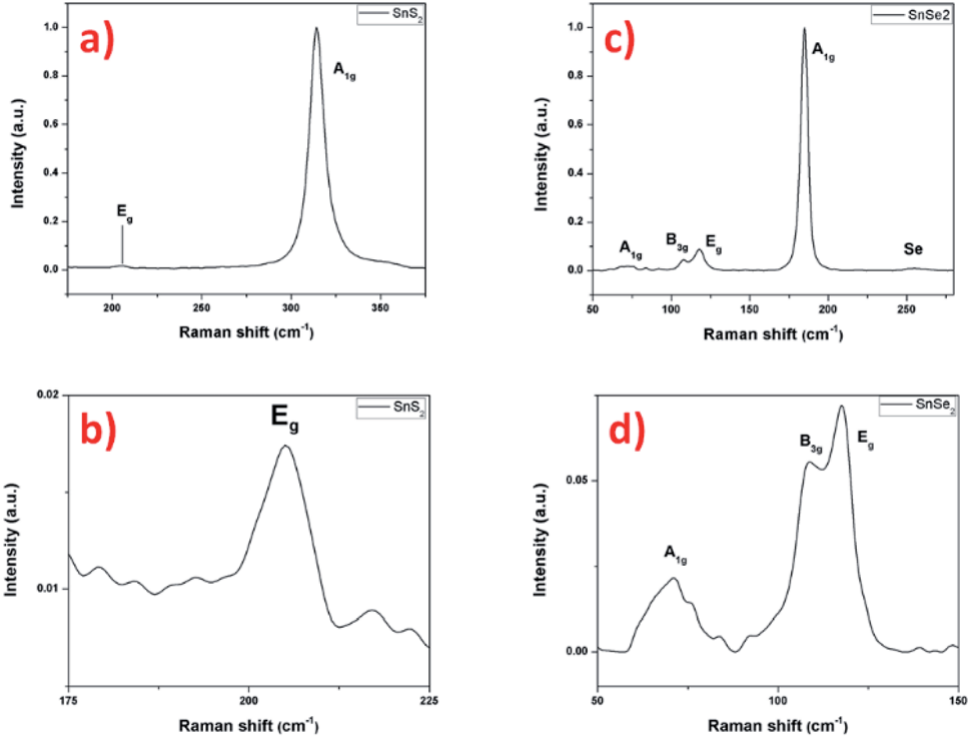
Raman spectrum with 532 nm excitation laser of SnS_2_ nanosheets (a and b) and SnSe_2_ nanosheets (c and d).

The Raman scattering measurement was done to study the signature of vibrational modes to confirm the formation of the SnS_2_ nanosheets. Fig. 8a shows the Raman spectrum of SnS_2_ nanosheets with two main peaks of 205 and 315 cm^−1^, assigned to the phonon modes. The most intense peak at 315 cm^−1^ is assigned as A_1g_ mode corresponding to vertical out-of-plane vibration between S–S and in the Fig. 8b the E_g_ correspond to non-degenerate in-plane vibration mode with a single band at 205 cm^−1^.^11,50^ The low intensity of the E_g_ peak is explained by the low thickness of the nanosheets, reducing the in-plane scattering centers as already reported.^51^ The Raman signature of the SnS_2_ material agrees with the previous results and confirms the crystallization into hexagonal 2H structure.

In the Fig. 8c, the Raman spectrum shows an intense peak that is assigned to the A_1g_ mode at 184 cm^−1^ and another very weak peak corresponding to E_g_ mode at 117 cm^−1^, signature of the SnSe_2_ material.^52,53^ The magnification of the E_g_ peak region surprisingly shows two small additional peaks at 70 cm^−1^ and 108 cm^−1^. These peaks cannot be definitively attributed to SnO_2_, as localized Raman modes on sputtered thermally oxidized tin metal were reported at different positions (typically at 112 cm^−1^ and 210 cm^−1^ for oxidation temperatures up to 400 °C with the appearance of further peaks at higher temperatures).^54^ Alternatively, the presence of another structure such as SnSe can be envisaged, this structure is characterized by four intense peaks in the region considered, localized at about 70, 108, 130 and 149 cm^−1^, related to A^1^_g_, B_3g_, A^2^_g_ and A^3^_g_, respectively.^55,56^ Here, only two weak peaks are well-visible that could be attributed to the A^1^_g_ and B_3g_ modes to the SnSe material. The other two can be guesstimated in the background noise. Finally, the broad peak located at 255 cm^−1^ can be attributed to the presence of amorphous Se.^57^ This had already been reported and explained by a condensation phenomenon of Se during cooling.^58^

**Fig. 9:**
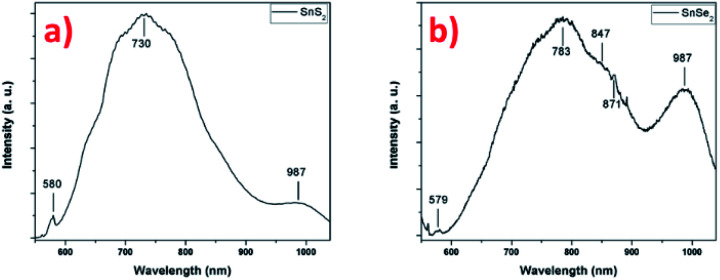
PL spectra of (a) SnS_2_ nanosheets and (b) SnSe_2_ nanosheets.

The photoluminescence of the two tin-based nanomaterials was studied at the excitation wavelength of 532 nm in Fig. 9. Fig. 9a shows the PL spectrum the SnS_2_ sample exhibiting different features: a low intensity peak at 580 nm (2.14 eV) that is attributed to the indirect bandgap of thin SnS_2_ film^59,60^ and a high intensity broad peak centered at 730 nm (1.70 eV) whose origin can have several sources according to the literature. Firstly, the presence of another nanomaterial, for example SnS nanosheets, giving rise to a PL emission at this energy, despite the fact that other techniques indicate the sole presence of 2H-SnS_2_. However, the energy does not correspond to the band gap of SnS around 825 nm (1.5 eV) and the large peak width is not characteristic of a single and well-defined energy transition.^61^ A report on 2D flakes of SnS_2_ synthesized by a wet chemical synthesis technique, shows similar features on the PL spectrum (at 532 nm) with a sharp peak at about 580 nm (indirect excitonic peak as previously assigned) and a broader peak centered at about 660 nm.^11^ The authors associated this transition to indirect exciton recombination of 4H phase of SnS_2_ present in the sample, rejecting the other possibilities due to the high quality of their synthetized SnS_2_ flakes (no impurities, no sulfur vacancies, *etc.*). In our SnS_2_ nanosheets, the 4H phase is not observed, what suggests another origin for this broad peak such as effect of crystalline defects/impurities or effect of interface strain between film and substrate, generating bound excitons recombination.^59,62^ Actually, it was reported that compared to the PL of bulk SnS_2_ (centered at 532 nm), the PL of exfoliated SnS_2_, shows a broad peak centered at 680 nm dramatically associated to electron–phonon interaction caused by the large amount of sulfur vacancies.^63^ Considering our SnS_2_ nanosheets, the structural and chemical characterizations demonstrates that high crystalline nanosheets of SnS_2_ have been successfully synthetized and the presence of impurities or vacancies were not observed. However, it was reported for transition metal dichalcogenide materials that bulk characterization techniques such as XRD cannot give a perfect overview of the quality of the synthesized material, which techniques such as Raman and PL spectroscopy do.^64^ The possibility of having vacancies/defects in the samples cannot be excluded, but due to the relative thickness of nanosheets such information is very hard to get by TEM. In addition, morphology may also influence the PL results. The nanosheets are oriented vertically with respect to the substrate, exposing their edges. Indeed, it has been shown that there is an increase in photoluminescence at the edges and also at the grain boundaries of triangular WS_2_ monolayers, explained by the different structure and composition of these regions compared with the center.^65,66^ A related study demonstrated a larger monosulfur vacancies concentration near the edges in comparison with the interior, explaining the features observed in PL.^67^ In agreement, in MoS_2_, exposed edges of the nanosheets due to the vertical orientation have been associated with enhanced PL emissions.^41^ To confirm this hypothesis, we performed PL experiments on a SnS_2_ sample with a different morphology (*i.e.* spheres of around 500 nm in diameter composed of small nanosheets (flower-like nanostructure, without any preferential alignment)) and compared with the result obtained on the vertically aligned SnS_2_ nanosheets sample (Fig. S8†). Compared to the PL spectrum recorded on the flower-like nanostructure, the PL of the vertically aligned sample is enhanced the hypothesis that the morphology plays a rule due certainly to the number of exposed edges. The conclusion we can draw from these photoluminescence experiments is that structural defects are certainly present at the edges of the nanosheets, explaining the origin and energy position of the high-intensity broader peak and the enhancement of the PL. The peak observed at 987 nm (1.26 eV) in the Fig. 9a, may be attribute to the emission signal of the substrate (Fig. S9†), in this case we used SiO_2_ as substrate.

Fig. 9b presents the PL spectrum of the SnSe_2_ sample. The first peak at 579 nm (2.15 eV) could be attributed to the direct allowed transition in SnSe_2_, value close to ones given in the literature.^68^ This value is close to one given for a monolayer and it is in agreement with the one reported for 50 nm film.^69^ The second broad and more intense peak located at 783 nm (1.58 eV) presents a well-defined shoulder at 847 nm (1.46 eV). For this material, few results have been reported on photoluminescence studies. However, due to the similarity of the results with the SnS_2_, similar conclusion could be drawn to explain the PL spectrum with the possible presence of defects/vacancies and the edges effect due to the vertical orientation of the nanosheets. A notable difference is the presence of a shoulder located at 847 cm^−1^ that may be associated to the presence of another phase, already detected in Raman spectroscopy. Indeed, the formation of polycrystalline thin films of SnSe and SnSe_2_ has been reported for low selenization temperature (<470 °C), hexagonal SnSe_2_ is formed and for higher temperature (<530 °C) orthorhombic SnSe become the preferential material PL.^58^ The authors showed for the material synthesized at 470 °C made of SnSe_2_, the presence of SnSe phase with a bandgap value of 1.43 eV (ref. 58) that is the close value found in our case with 1.46 eV. Surprisingly, this value does not correspond to a gap value for orthorhombic SnSe but rather to that of cubic SnSe.^70^ The other intense peak observed at 987 nm (1.26 eV) can be attribute, as previously shown for SnS_2_, to the photoluminescence emissions of the substrate. In this case, the signature of the substrate can also be found in the low intensity peak at 871 nm (1.42 eV) in the shoulder of the more intense and broader peak (see Fig. S9† for comparison). These results indicate that the SnSe_2_ sample is less homogeneous than SnS_2_ sample (as we can see in the Fig. 1) and the transitions of the substrate are more visible (Fig. S9†).

**Fig. 10:**
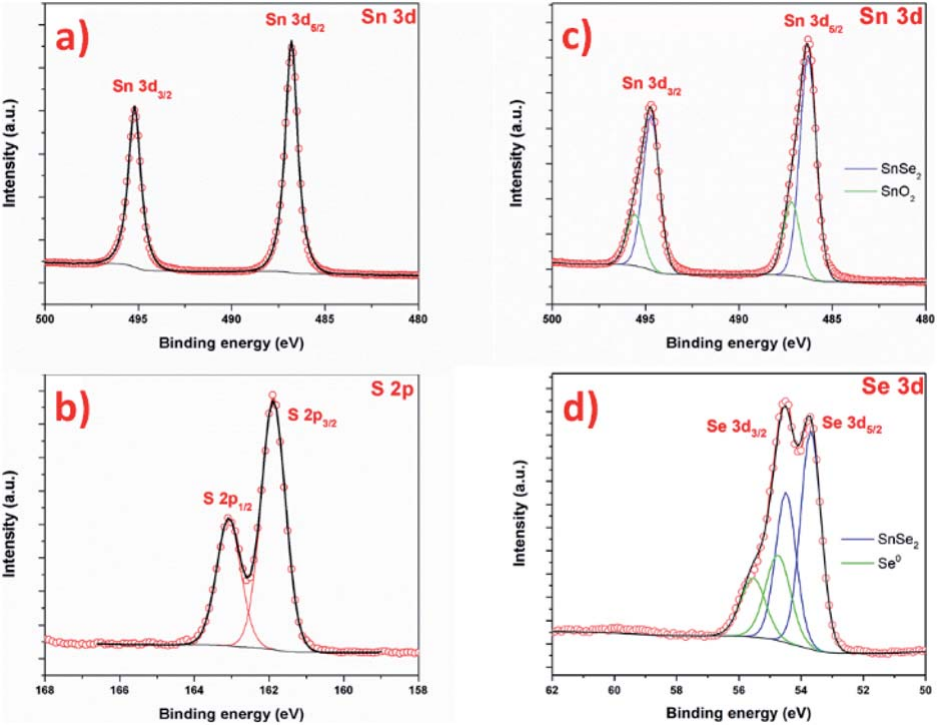
XPS spectra of the SnS_2_ nanosheets (a) Sn 3d and (b) S 2p and SnSe_2_ nanosheets (c) Sn 3d and (d) Se 3d.

The chemistry of the as-prepared products was further investigated by XPS as shown in Fig. 10. Fig. 10a and b present the Sn 3d and S 2p core level of SnS_2_ samples. The Sn 3d spectrum is composed of one doublet, with contributions centered at 487.1 and 495.6 eV, attributed to Sn 3d_5/2_ and Sn 3d_3/2_ of Sn^4+^ in SnS_2_, respectively.^15,71^ No other contribution is observed.^72^ The S 2p spectrum (Fig. 10b) is composed of a one doublet, with S 2p_3/2_ and S 2p_1/2_ signal centered at 161.8 and 162.7 eV respectively, attributed to S^2−^ species in SnS_2_.^61^ Both Sn 3d and S 2p confirms the successful formation of SnS_2_ phase, without another phase. The Fig. 10c and d shows Sn 3d and Se 2p signal of SnSe_2_ sample. The Sn 3d is composed of a first doublet, with contributions centered at 486.3 eV and 494.7 eV, attributed to Sn 3d_5/2_ and Sn 3d_3/2_ states of Sn^4+^ in SnSe_2_. A second doublet is observed, with contributions at 487.2 and 495.6 eV. The Se 3d spectrum is composed of a first doublet with contributions centered at 53.7 eV and 54.5 eV attributed to Se 3d_5/2_ and Se 3d_3/2_ of SnSe_2_ phase.^52^ Here also, a second doublet is observed, with Se 3d_5/2_ and Se 3d_3/2_ contributions centered at 54.8 eV and 55.5 eV respectively. The interpretation of selenium XPS signal is complex and the literature about XPS of SnSe_*x*_ reports contradictory and wrong interpretation of Se and Sn signals. One can cite the absence of Se 3d_5/2_ contribution in SnSe^73^ or an area of 3d_3/2_ contribution higher than the 3d_5/2_ in ref. 74. Here, the contribution Sn 3d at 487.2 is attributed to Sn oxide. In reference paper, SnO_2_ is reported at 487.8 in ref. 75, 486.8 in ref. 76 and 486.3 eV.^77^ The observation of Sn oxide contribution is consistent with previous results: tin oxide is formed prior to the selenization (and sulfurization), and SEM images show that some area is not covered by the nanosheet, namely the XPS also probes this surface. For SnS_2_, the covering is more uniform, certainly due to the total reaction of SnO_2_ with S. In this case, explaining that the oxidation is not observed by XPS. Concerning the second contribution in Se signal, at 54.8 eV, this contribution is not fully understood in the literature. If this contribution could be attributed to SnSe phase, in agreement with,^36^ one should also see a contribution in the Sn signal, at lower binding energy (shifted by ≈ −0.7 eV). The Se contribution cannot be attributed to SeO_2_, as this contribution is centered at much higher binding energy, around 59.5 eV.^75,78^ Alternatively, the formation of SnSe_2−*x*_ metastable phase can be considered. Indeed, D'Olimpio *et al.*^75^ did a careful investigation of SnSe_2_ chemistry when exposed to different environment. However, this would imply also a contribution on the Sn signal, and this contribution would be between SnSe_2_ and SnSe, *i.e.* at a lower binding energy of 483.3 eV for the Sn 3d_5/2_ contribution of this metastable phase. The most probable explanation would lie in the presence of metallic selenium (Se^0^). The Se 3d_5/2_ signal of metallic selenium is reported in higher binding energies than tin selenide materials at 56.0 eV for Lee *et al.*^79^ and at 54.7 eV by Vishwanath *et al.*,^80^ this latter value is in good agreement with our data and would confirm the Raman analysis.

We have demonstrated that vertically aligned nanosheets of SnS_2_ and SnSe_2_ have been synthesized using a simple and scalable method *via* a CVD technique using direct reaction of vapor phase (sulfur or selenium) on deposed tin films.

## Discussion

With the results discussed above, a possible growth model for the SnS_2_ and SnSe_2_ aligned nanosheets prepared by using sulfurization and selenization, respectively, of pre-deposited 50 nm Sn films *via* magnetron sputtering is proposed. The morphologies of the tin based material produced by this method seems to be influence by different parameters, however the precursor film is the key parameter.^38^ Indeed, it has been shown that the magnetron sputtering parameters play an essential role in the morphologies of the deposited films, especially the argon pressure.^38^ By example, it has been reported that in tungsten deposit, a lower argon pressure results in a denser film structure than with a higher pressure where the film has a porous structure with cracks. The resulting tungsten selenide film results in different sheet orientations either parallel to the substrate for dense tungsten films (low argon pressure) or perpendicular to the substrate for porous films (higher argon pressure).^38^ Here, the film deposition has been made at 1 × 10^−3^ mbar at room temperature that is a lower value than ones reported in ref. 38 and the chemistry of the film is different, tin instead of tungsten, however the results obtained seem to follow the same trend, where the vertical alignment of the nanosheets is not observable with direct reaction (sulfurization or selenization) on the film thus prepared, certainly due to the film density. The effect of the deposition pressure on the sputtering tin films has been not studied. To overcome this problem, the solution found was to oxidize the tin film as of homogeneity is promoted. Indeed, the sputtered film is metallic but when it is transferred on air after the deposition a partial oxidation occurs due to the thickness of the film. For a homogeneous oxidation of the whole film, an oxidation step is performed. Depending on the level of oxidation, the surface will change determining the diffusion of the S/Se, which is very important for the vertically growth. Indeed, we observed that the morphology of the nanostructures strongly depends on the oxidation level before sulfurization/selenization. As an example, for sulfurization: (i) the vertical alignment of SnS_2_ nanosheets was not observed on tin film as sputtered (with partial oxidation in air); (ii) samples synthesized on tin film oxidized at 200 °C for 2 hours with air flow presents a flower-like morphology with short nanosheets grouped in the form of spheres without any preferential alignment; and (iii) samples synthesized on tin film oxidized at 300 °C for 2 hours with air flow shows a vertical orientation of the nanosheets with uniformly distribution in all the surface. Therefore, it can be postulated that depending on the level of oxidation, different thickness of the film will be oxidized and this will determine the diffusion of S/Se, a very important factor for vertical growth of nanosheets. Indeed, it has been reported that the diffusion of S/Se through the film is necessary and that this process is the limiting factor for the orientation of the nanosheets.^40^ According to our experimental data, the sulfur diffusion increases with the thickness of the oxidized layer, it seems that S interacts just with Sn near the film surface for case (ii) and short nanosheets are formed, while for case (iii) due to the higher temperature the Sn diffuses deeper leading to a pronounced change of the surface morphology. Another parameter reported in the literature playing an important role in the final product characteristics is the composition of the reactive atmosphere near the film, more or less rich in S/Se.^81^ Indeed, it has been shown for MoS_2_ that in order to have a complete reaction with the oxidized film, it is necessary to have an atmosphere rich in S for this to be the dominant mechanism, instead of having an atmosphere deficient in S, as in this case the modification of the surface occurs due to the dominant mechanism of oxide segregation/coalescence.^81^ The effect of the sulfur concentration during the synthesis was not evaluated, however we can assume that the atmosphere is rich enough in S/Se for the sulfurization/selenization reaction to occur. Therefore, the Sn oxide film is reduced by sulfur or selenium and we obtained SnS_2_ or SnSe_2_ in a short synthesis time. A very fast reduction of Sn oxides by S/Se might have an important role in the formation of vertically aligned layered nanosheets. The growth of nanosheets, occurs along two directions ([001] and [100]), with higher growth velocity along [001] leading to the formation of sheet-like structures. The vertical alignment of nanosheets formation is possibly favored by its anisotropic atomic bonding nature. The differences in the reactivity of sulfur and selenium plays an important role in the syntheses of the SnS_2_ and SnSe_2_ nanomaterials. Certainly, Se chemical reactivity is much lower than S one and a suitable reducer agent is required.^82^ For that, it is necessary the addition of H_2_ to promote the formation of H_2_Se which in turn provides atomic Se for the formation of SnSe_2_,^58^ because without H_2_ the growth of SnSe_2_ does not occur. Furthermore, the presence of hydrogen can also lead to the reduction of the oxidized tin film, which is not the goal. However, by optimizing the amount of hydrogen introduced, the selenization reaction occurs to produce vertically aligned layered SnSe_2_ nanosheets. We perceived that while during the synthesis of SnSe_2_, SnSe was formed, in the case of SnS_2_, the SnS was not observed. This can be explained by the thermodynamic conditions of the reaction. It has been shown that the reaction to form SnS is not spontaneous in an inert atmosphere as in our case where only argon is introduced during the reaction. If hydrogen had been used during the synthesis, the SnS material could have been synthesized as it requires a reducing atmosphere.^51^ Concerning the selenium material, the presence of hydrogen is necessary as described above to obtain the correct selenium reactive species, allowing the formation of the different selenium materials. A last parameter to consider is the temperature. It has been shown that temperature plays a major role in phase selection between SnSe and SnSe_2_,^36,58^ a lower selenization temperature allows the formation of a dominant SnSe_2_ phase, whereas a higher temperature leads to the formation of the SnSe phase (this is also dependent on other parameters such as the gases in the reaction chamber). With an intermediate temperature, a mixture of the two phases can be observed.^58^ Our reaction occurs at 450 °C, which is a relatively low selenization temperature in the range of temperatures reported for the synthesis of the SnSe_2_ phase, therefore explaining the observation of a mix of both phases, as we reported in this work. This temperature is not far from the intermediate temperature between the SnSe_2_ and SnSe formation temperatures, which explains the fact that domains of the other SnSe phase can be found in a product composed mainly of SnSe_2_. This fact have been already reported in ref. 58 for a selenization temperature of 470 °C. Finally, for such syntheses, the temperature is relatively low compared to the formation temperatures of other metal dichalcogenides, which may also explain the presence of structural defects (vacancies).

## Conclusions

We have demonstrated the synthesis of vertically aligned SnS_2_ and SnSe_2_ nanosheets. This was achieved using a simple one-step CVD technique by direct sulfurization/selenization of a pre-oxidized tin film. We evidenced the quality of the as-synthesized materials by employing XRD, Raman spectroscopy, XPS and electron microscopies. Vertical and homogeneous alignment of well-crystalline 2H-phase SnS_2_ nanosheets have been demonstrated, the nanosheets, however, having structural defects at their edges. The SnSe_2_ nanomaterial has the same form as the SnS_2_ nanomaterial, *i.e.* vertical alignment and well-crystalline 2H phase with also structural defects at their edges, however with some differences were observed: the nanosheets are larger, less homogeneity, presence of another nanomaterial SnSe as well as unreacted selenium. The growth mechanism is discussed on the basis of our results.

## Author contributions

A. S.-C. and J.-F. C. conceived, and carried out the experiments. E. H. contributed to XPS measurements and analysis. S. A. and C. B. contributed to the Raman and PL measurements. R. A. contributed to STEM and EDS measurements and analysis. A. S.-C. and J.-F. C. made the analysis of the data. A. S.-C. and J.-F. C. wrote the manuscript. All of the authors provided critical feedback and helped shape the research and analysis. All authors have read and agreed to the published version of the manuscript.

## Conflicts of interest

The authors declare that they have no competing interests.

## Supplementary Material

RA-011-D1RA05672G-s001
