# Supplementary material for: Atmospheric pressure chemical vapor deposition growth of vertically aligned SnS2 and SnSe2 nanosheets
Source: RSC Adv. 2021 Nov 11;11(58):36483–93. doi: 10.1039/d1ra05672g (PMC9043430; doi:10.1039/d1ra05672g)
Supplement: RA-011-D1RA05672G-s001 [file RA-011-D1RA05672G-s001.pdf]

Electronic Supplementary Material (ESI) for RSC Advances.
This journal is © The Royal Society of Chemistry 2021

Supplementary Information

Figure S1.

The influence of the tin film oxidation temperature in the morphology of the sample (Figure S1). The tin film oxidation was performed in air before synthesis. When is pre-oxidized at 200 °C the final morphology presents nanospheres with some nanosheets (Figure S1 a), for the optimal temperature (300 °C) only nanosheets are observed (Figure S1 b). For oxidation performed at temperatures higher than the optimal one, smaller nanosheets not-well distribute are formed (Figure S1 c).

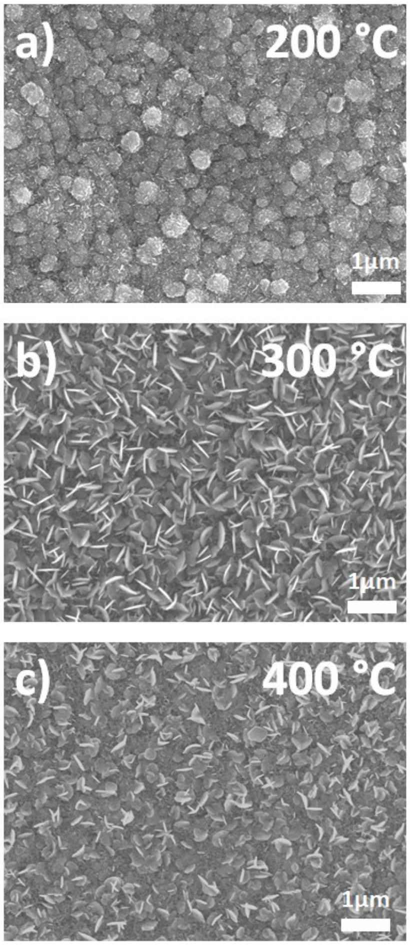

Figure S1. Influence of the Sn film oxidation temperature on the morphology of SnS_2 nanosheets a) at 200 °C, b) at 300 °C and c) at 400 °C.

Figure S2.

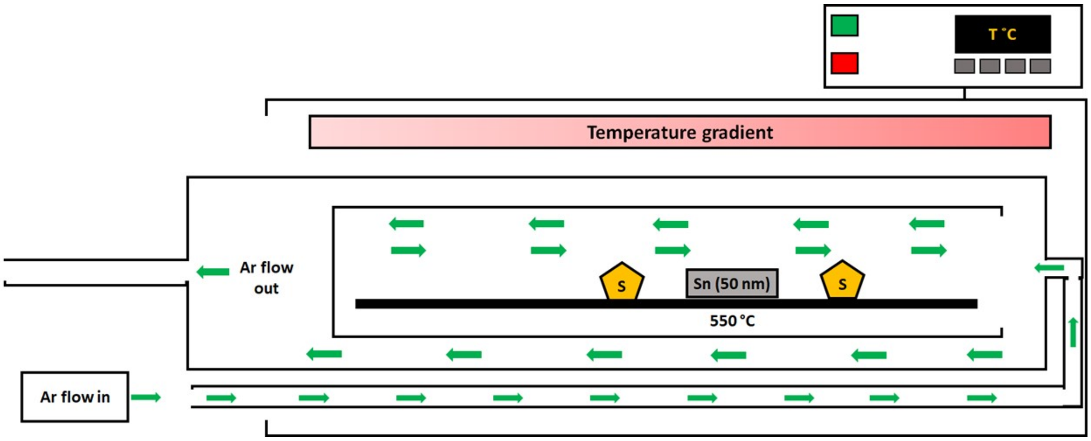

Figure S2. Schematic diagram of the CVD method for the synthesis of SnS_2 nanosheets.

Figure S3.

Figure S3 shows the influence of the synthesis temperature in the morphology of the SnS_2 nanosheets. For synthesis at 450 °C (Fig. S3 a) a non-homogenous sample with some nanosheets is obtained. Similar morphology is obtained for synthesis at 500 °C (Fig. S3 b) and at 600 °C (Fig. S3 c) in which a larger number of sheets with length in the scale of μm is observed. For higher temperature (650 °C) the metal film is partially destroyed (Fig. S3 d).

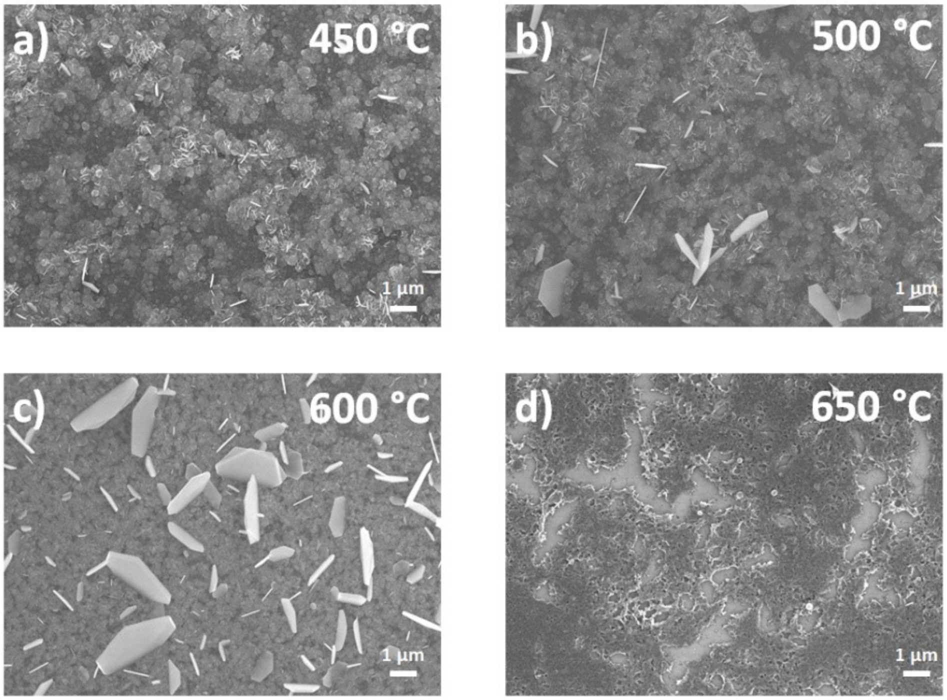

Figure S3. SnS_2 morphology for different synthesis temperatures a) at 450 °C, b) at 500 °C, c) at 600 °C and d) at 650 °C.

Figure S4.

Following the selection of the optimal synthesis temperature, it was important to determinate the optimal carrier (Ar) gas flow. Figure S4 shows the influence of the gas carrier flow during the synthesis of SnS_2. For high gas flow (Figure S4 a) the final product presents nanosheets with different length ranging from few nanometers to microns, the high concentration of Ar affected the diffusion of the S atoms into the metal film. Decreasing the Ar flow to 70 sccm, the nanosheets are homogeneous in size and distribution (Fig. S4 b), the optimal gas flow favorizes the better diffusion of the chalcogen into the tin sample. For Ar flow smaller than the optimal one (Figure S4 c) few nanosheets are formed; for a low Ar flow the availability of chalcogen atoms for the formation of the nanosheets is reduced preventing their formation.

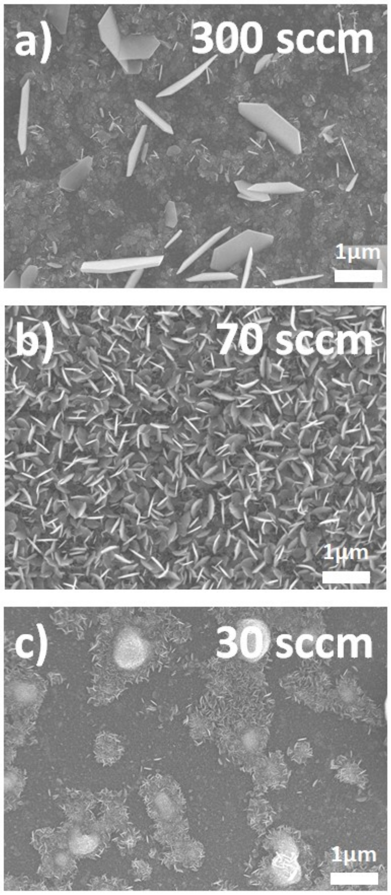

Figure S4. Influence of the carrier gas used in the synthesis of SnS_2 nanosheets in the morphology of the sample a) 300 sccm, b) 70 sccm and c) 30 sccm.

Figure S5.

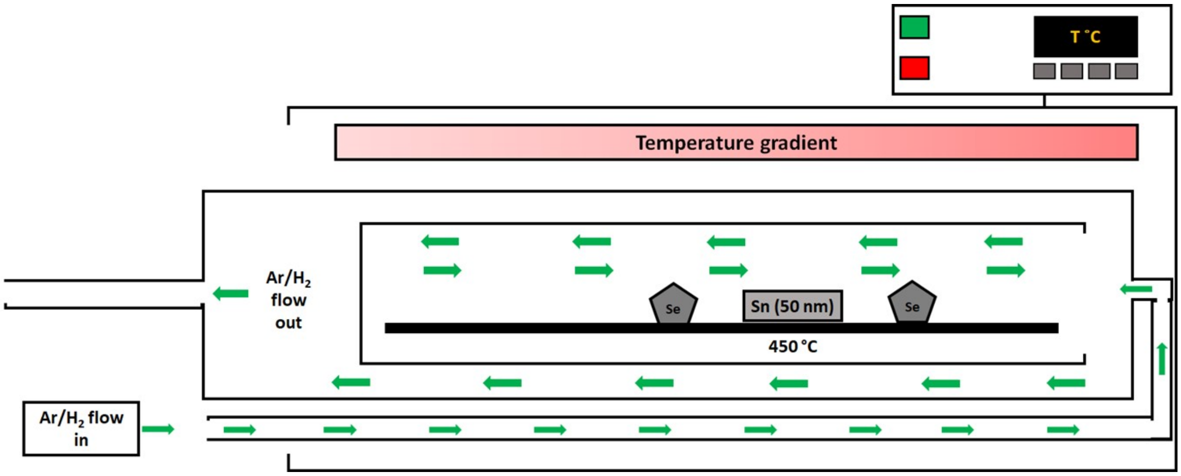

Figure S5. Schematic diagram of the CVD method for the synthesis of $SnSe_2$ nanosheets.

Figure S6.

Figure S6 shows the influence of the synthesis temperature in the morphology of the $SnSe_2$ sample. The SEM image of the sample synthesized at 550 °C (Fig. S6 a) shows the formation large nanosheets, reducing the temperature 50 °C (Fig. S6 b), it can be observed a considerable change in the size of the nanosheets with simultaneously increase in its number. In Figure S6 c) reducing the synthesis temperature to 450 °C, few vertically-aligned nanosheets can be observed.

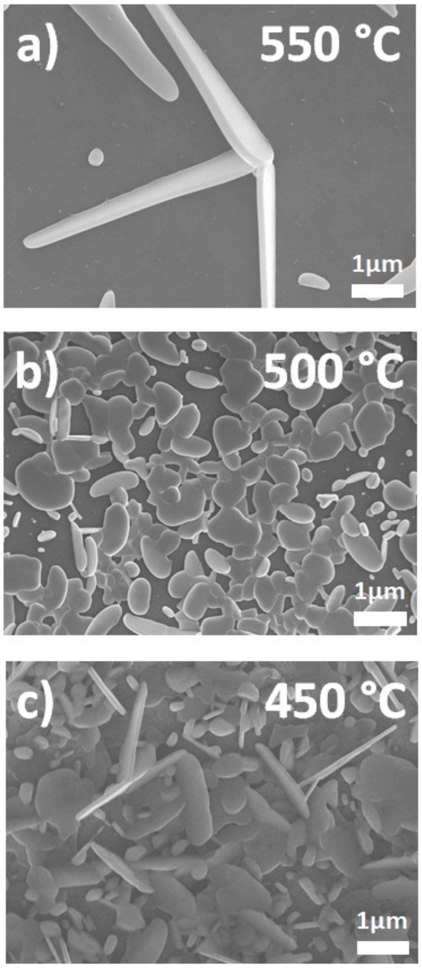

Figure S6. Effect of the synthesis temperature in the morphology of the $SnSe_2$ nanosheets a) at 550 °C, b) at 500 °C and c) at 450 °C.

Figure S7.

After the synthesis temperature was selected the next step, it would be determinate the gas flow. As it was explained in the article, H_2 is used in this synthesis due to the presence of a strong reducer is indeed mandatory for the selenization reaction, compared with the sulfurization reactionand for that it was important find the good balance between the two-carrier gas during the synthesis. Figure S7 show the influence of the gases flow. Using 70/5 sscm of Ar/H_2 (Figure S7 a) during the synthesis few large platelets are formed. Decreasing the Ar flow (40/5) the smaller platelets were formed (Figure S7 b). Finally, reducing the flow to (10/10 of Ar/H_2) the substrate was entirely covered with platelets and aligned nanosheets (Figure S7 c).

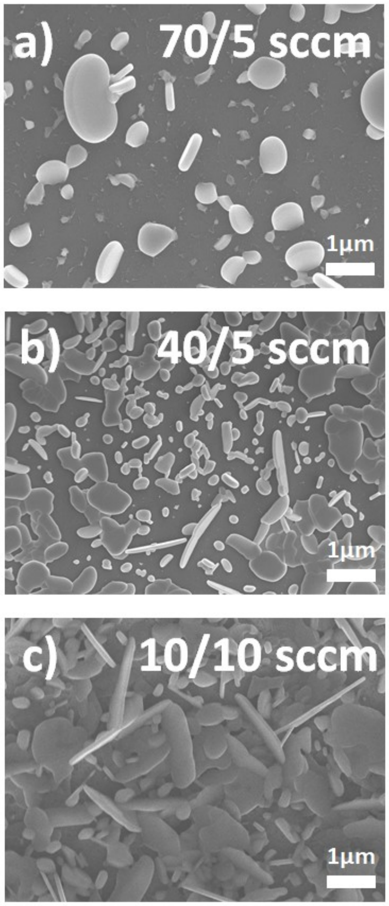

Figure S7. Influence of Ar/H_2 flow in the morphology of the $SnSe_2$sample a) 70/5 sccm, b) 40/5 sccm and c) 10/10 sccm.

Figure S8.

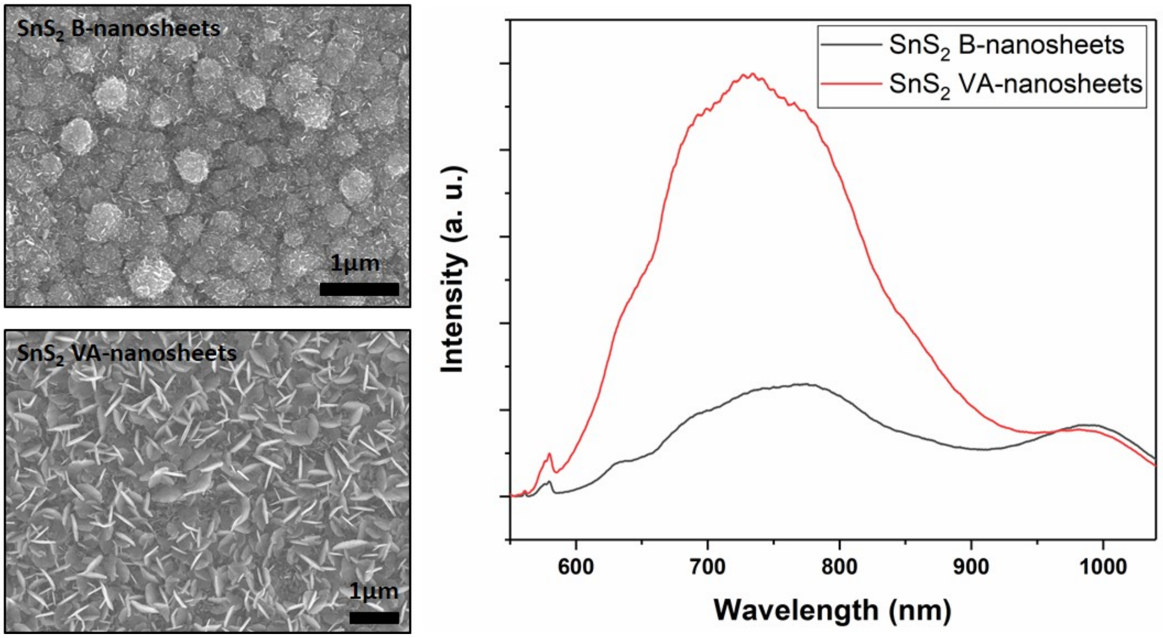

Figure S8. SEM and PL comparison of SnS_2 with different morphologies.

Figure S9.

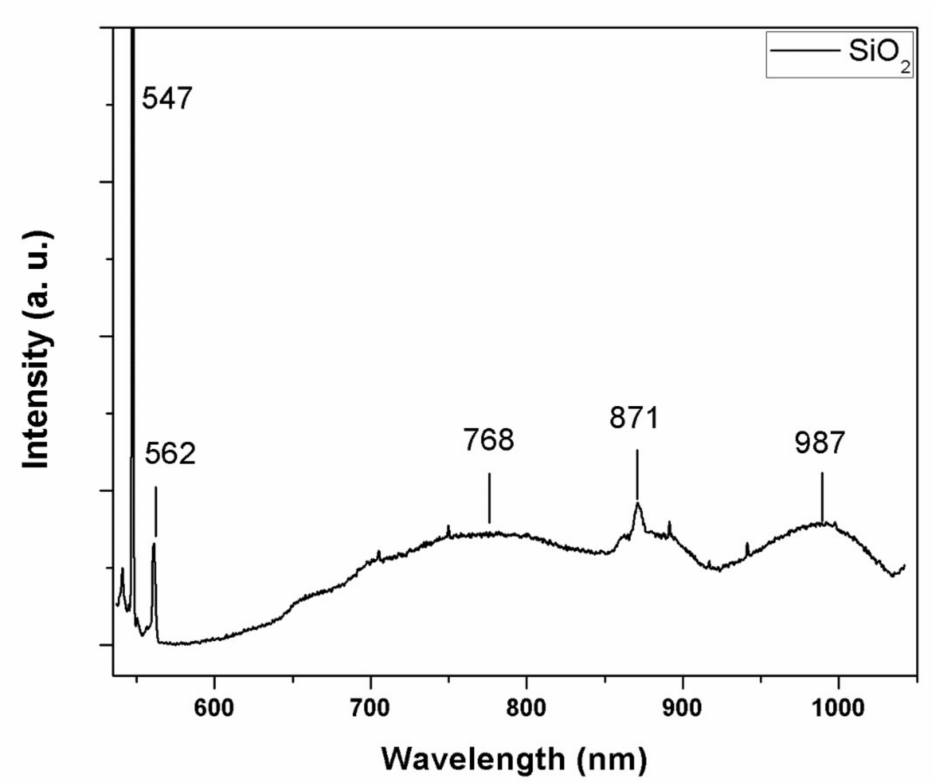

Figure S9. PL spectra of SiO_2
